# The Relationship between the Source of Dietary Animal Fats and Proteins and the Gut Microbiota Condition and Obesity in Humans

**DOI:** 10.3390/nu15143082

**Published:** 2023-07-09

**Authors:** Wojciech Kazura, Katarzyna Michalczyk, Dominika Stygar

**Affiliations:** 1 Department of Physiology, Faculty of Medical Sciences, Medical University of Silesia, Jordana Street 19, 41-808 Zabrze, Poland; 2 SLU University Animal Hospital, Swedish University of Agricultural Sciences, SE-750 07 Uppsala, Sweden

**Keywords:** dietary fats, dietary patterns, dietary protein, gut microbiome, obesity

## Abstract

The relationship between gut microbiota and obesity is well documented in humans and animal models. Dietary factors can change the intestinal microbiota composition and influence obesity development. However, knowledge of how diet, metabolism, and intestinal microbiota interact and modulate energy metabolism and obesity development is still limited. Epidemiological studies show a link between consuming dietary proteins and fats from specific sources and obesity. Animal studies confirm that proteins and fats of different origins differ in their ability to prevent or induce obesity. Protein sources, such as meat, dairy products, vegetables, pulses, and seafood, vary in their amino acid composition. In addition, the type and level of other factors, such as fatty acids or persistent organic pollutants, vary depending on the source of dietary protein. All these factors can modulate the intestinal microbiota composition and, thus, may influence obesity development. This review summarizes selected evidence of how proteins and fats of different origins affect energy efficiency, obesity development, and intestinal microbiota, linking protein and fat-dependent changes in the intestinal microbiota with obesity.

## 1. Introduction

The gut is inhabited by about 100 trillion organisms, including 35,000 species of bacteria [1], that create a specific ecosystem that helps maintain the health of the intestines, as well as the entire individual [2]. The importance of gut microbiota is well documented [3,4], and increasingly, the role of the gastrointestinal tract in the etiology and influence on treating metabolic disorders, such as obesity, type 2 *diabetes mellitus* (T2DM), and cardiovascular diseases (CVD), is emphasized [1,2]. One of the pathophysiologic links between obesity and gut microbiota is a leaky gut syndrome. The gut mucosa in leaky gut syndrome can trigger an inflammatory response that enables the development of peripheral insulin resistance. Moreover, the leaky gut syndrome may result from the disturbed secretion of tissue hormones responsible for the satiety feeling and the non-digestible food ingredients influencing the gastrointestinal microbiota. The leaky gut syndrome manifests in impaired glucose uptake and utilization in skeletal muscles, intensified fat deposition in adipose tissue, and an increased release of free fatty acids. In consequence, hyperglycemia increases, and the non-alcoholic fatty liver disease (NAFLD) develops [5]. Nevertheless, the increased consumption of high-calorie foods and increased total energy consumption are possibly major factors responsible for the obesity epidemic [6,7]. Dietary patterns play an important role in obesity development [8] and in shaping the intestinal microbiota in humans [9,10,11,12,13,14,15].

## 2. Materials and Methods

The systematic literature research was conducted using the *Pubmed* and *Google Scholar* databases. The following keywords were used individually or in combination: obesity, gut microbiota, large intestine, colon, diet, fat, and proteins. The database search focused on original research and review articles presenting studies conducted on animals and humans, published between May 1974 and February 2023. The non-English publications were excluded from the search results.

## 3. Healthy Human Intestinal Microbiota

The basic assumption regarding changes in the composition of the intestinal microbiota in metabolic disorders states is the knowledge about the composition and function of the intestinal microbiota in metabolically healthy people. However, the normal human intestinal microbiota has not been thoroughly defined regarding its taxonomic diversity. The relative distribution of gut bacteria and archaea is partially unique to individuals in terms of microbial species differences, microbial development [16], and genome [17,18]. The environment and lifestyle also influence the diversity of the human gut microbiota [19]. However, healthy individuals generally are characterized by a high diversity of taxa, high genetic diversity of microorganisms, and a functionally stable microbiome [18]. However, it is worth noting that the gut bacteria’s high diversity and species richness are not objective indicators of a healthy microbiome, since intestinal transit time also affects the richness of microorganisms [20]. Extended transit time may increase species diversity but not necessarily in a healthy gut microbiota. In the past, most of the knowledge on adult human gut microbiota was derived from labor-intensive methods based on culturing bacterial cultures [21]. The ability to study intestinal microbiota has improved significantly due to the emergence of high-throughput and low-cost methods for genetic code sequencing. The identification of the bacterial 16S rRNA gene is a popular approach [22,23], because this gene is present in all bacteria and archaea and contains nine highly variable regions (V1–V9), which allows distinguishing the species easily. Early studies using the entire 16S rRNA gene sequencing emphasized the extreme insensitivity and bias of culturing methods, as 76% of the rRNA sequences indicated new and uncharacterized species [24]. Recently, the emphasis on 16S rRNA sequencing has shifted to the analysis of shorter gene sub-regions [23], but it can also be misleading [22]. It seems that genome-wide metagenomics can provide more reliable estimates for the composition and diversity of microbiota [22]. So far, data from the MetaHit and Human Microbiome projects have produced the most comprehensive picture of the microbial spectrum associated with the human gut microbiota [25,26]. The data collected in these studies allowed for identifying 2172 species isolated from humans, classified into 12 groups, 93.5% of which were *Proteobacteria*, *Bacteroidetes, Firmicutes*, and *Actinobacteria*.

Three out of the twelve groups contained only one species isolated from humans, including the intestinal species *Akkermansia muciniphila*, the only known member of *Verrucomicrobia* sp. In humans, 386 of the identified bacterial species are strictly anaerobic, and their number varies depending on the part of the body. Lower concentrations of anaerobes are observed on the skin, in the mouth, or in the vagina, while higher concentrations are observed in the intestine. In addition, the concentration of anaerobes increases along the digestive tract, from the stomach to the colon [25]. The gut microbiota is diverse and exhibits high functional redundancy [27,28]. Schluter et al. [27] combined 249 new and 1018 already published bacterial sequences and, based on 9,879,896 genes, estimated the functional capacity of the human gut microbiome [27]. They identified the presence of country-specific microbial signatures, suggesting that the gut microbiota composition is influenced by environmental factors such as diet and possibly also by host genetics [27,29]. However, the microorganisms that differ in composition may show a certain degree of functional redundancy, resulting in a similar profile of proteins or metabolites [29]. This information is critical to the researchers developing therapeutic strategies to modify and profile microbes in disease states.

## 4. Development of the Human Gastrointestinal Microbiota

It is generally believed that the human gastrointestinal microbiota development begins at birth, although some studies reported microbes presence in uterine tissues, placenta, chorionic villi, and amniotic fluid [30,31,32]. The newborn’s digestive tract is rapidly colonized at or shortly after birth, and life events such as disease, medication usage—in particular, antibiotics—and dietary changes cause chaotic changes in the microbiota [31,33,34]. A key determinant of the neonatal microbiome is the route of delivery [35]. The microbiome of naturally born infants contains a large number of *Lactobacilli* in the first few days of life, reflecting the high *Lactobacilli* load of the vaginal flora [36,37]. In contrast, the microbiota of infants born via cesarean section is depleted, and the colonization with *Bacteroides* sp. and facultative anaerobes such as *Clostridium* sp. is delayed [38,39]. It was also shown that 72% of vaginal infants have fecal microbiota similar to that of their mothers, while in cesarean-section infants, this percentage is reduced to just 41% [40]. Furthermore, the method of infant feeding is also of great importance for intestinal microbiota development. Exclusive breastfeeding is associated with a lower bacterial diversity and richness, lower abundance of *Bacteroides* and *Firmicutes*, and redirecting the microbial pathways to lipid and vitamin metabolism rather than carbohydrates [41]. Additionally, human milk oligosaccharides have an immunomodulatory effect and act as prebiotics in establishing the infant’s gut microbiota [42].

In the early stages of development, the microbiota is generally poorly differentiated and dominated by two major clusters, *Actinobacteria* and *Proteobacteria* [31,42]. It increases in the first year of life, when the microbial diversity develops and the microbiota composition approaches an adult-like microbial profile, with temporal patterns unique to each infant [43]. These changes result from introducing solid foods into the diet. The changes are particularly associated with an increase in the number of species related to *Bacteroidetes*, while the number of *Bifidobacterium*, *Lactobacillus*, and *Enterobacteriaceae* decreases [44]. By the middle of the third year of life, an infant’s microbiota composition, diversity, and functional capacity resemble that of an adult individual [31,33]. Although the composition of the intestinal microbiota in adulthood is relatively stable, it is still disturbed by various life events [45]. In people over 65 years of age, the bacterial flora changes: the *Bacteroidetes* phyla and *Clostridium* Phyla IV become more abundant compared to younger individuals, in which *Clostridium* Cluster XIVa predominates [46]. On the other hand, another study reported that the microbiota of younger and older participants (70 years of age) were relatively comparable, while the diversity of the centenarians’ microbiota was significantly reduced [47]. The microbiota of centenarians participating in the study also showed group-specific differences, such as an increase in facultative anaerobes (e.g., *Escherichia coli*) and a rearrangement of the profile of butyrate-producing species (e.g., a decline in *Faecalibacterium prausnitzii*) [47]. However, a study on the Chinese population showed that the fecal microbiota of active centenarians (94 years of age and above) did not differ significantly compared to middle-aged adults (30–50 years of age) [48]. In the elderly population, the microbiota diversity correlated with living conditions, such as community living or long-term home care [49]. Another study found that the ability of the microbiota to carry out metabolic processes, such as the production of short-chain fatty acids (SCFA) and amylolysis, is reduced in the elderly while, in turn, proteolytic activity is increased [50].

Given the increasing evidence on the role of SCFA as key metabolic and immune mediators, which promote the expansion and differentiation of regulatory T cells, it has been postulated that reduced SCFA levels may promote the development of intestinal inflammation in the elderly [51]. On the other hand, some studies show that the older adults had a greater functional potential for SCFA fermentation than young adults [52,53].

The chemical, nutritional, and immunological gradients along the gut also affect the density and composition of the gut microbiota. The small intestine usually presents high levels of acids and antimicrobial agents. The small intestine environment is aerobic, and the food content passes quickly [54]. These conditions limit bacterial growth, and only rapidly growing facultative anaerobes able to adhere to epithelium or mucus are thought to survive [55]. In mice, the microbial flora of the small intestine is largely dominated by *Lactobacillaceae* [56]. In turn, conditions in the colon favor a dense and diverse community of bacteria—primarily anaerobes-capable of using complex carbohydrates that are undigested in the small intestine. The colon is dominated by *Prevotellaceae*, *Lachnospiraceae*, and *Rikenellaceae* [54,56].

Contrary to the diverse composition of the microbiota in different sections of the gastrointestinal tract, the microbiota of different regions of the colon mucosa in the same individual is spatially homogeneous, both in terms of composition and diversity [57,58]. Conversely, stool/lumen and mucosa composition are variable [57,58]. For example, *Bacteroidetes* appear to be more abundant in stool samples than in the mucosa [57,59]. In contrast, *Firmicutes*, especially *Clostridium* XIVa, are more numerous in the mucous layer than in the lumen [59]. Interestingly, experiments on mice colonized by a variety of pathogen-free microbiota showed that the external ingrowth of the mucus of the large intestine creates a unique microbial niche. The bacterial species in the mucus showed differentiated proliferation and used more diverse resources than the same species in the lumen of the intestine [60]. These observations indicated that sampling methods should be carefully selected when analyzing the gastrointestinal microbiota composition.

Interindividual differences in the species and subspecies system of the intestinal microbiota outweigh the differences in the community system within the individual [57,61,62]. Earlier studies suggested the presence of ‘indigenous microbiota’—i.e., a set of the same abundant microorganisms in all individuals. However, it turns out that the microbial gene repertoire shows greater similarity between individuals than at the taxonomic level, which suggests that the basal microbiota may be better defined at the functional level than at the organism level [61]. More recently, individual microbiota differences have been classified according to ‘community types’ that predict changes and are environmentally dependent [63]. Multidimensional analysis of 33 samples of people of different nationalities revealed the presence of three enterotypes that can be identified based on differences in the level of one of the three types: *Bacteroides* (enterotype 1), *Prevotella* (enterotype 2), and *Ruminococcus* (enterotype 3) [64]. However, the evidence for the existence and formation of these enterotypes is controversial, as discussed in detail by Jeffery et al. [65].

## 5. The Role of the Digestive System Microbiota in the Host’s Energy Balance

The gastrointestinal microbiota plays a significant role in human health and disease [1]. Microbiota is involved in energy production and metabolic functions such as bile acid and choline metabolism, fermentation and absorption of undigested carbohydrates, or vitamins and exogenous amino acids supplementation [66,67]. Research shows that the gut microbiota can influence weight gain and obesity through several interconnected pathways, producing metabolites that influence inflammatory responses and modulating metabolic pathways and eating behaviors of the host through the gut–brain axis [68].

One of the essential metabolic activities of the microbiota is the production of non-volatile short-chain fatty acids (SCFAs) by fermentation of prebiotics—i.e., compound carbohydrates (e.g., resistant starches, pectins, gums, oligosaccharides, and plant cells), proteins, peptides, and glycoproteins [69,70,71]. SCFAs are fatty acids with fewer than six carbon atoms. The most important SCFAs are acetate, propionate, and butyrate [72]. The dominant SCFA-producing commensals are *Akkermansia muciniphilia*, *Prevotella* spp., *Ruminococus* spp., *Coprococcus* spp., *Faecalibacterium prausnitzii*, *Eubacterium rectale*, and *Roseburia* spp. [73]. Absorbable SCFAs modulate gut health and immune processes [74], hormone synthesis, and lipogenesis [75]. They also partake in numerous interactions with the host. Short-chain fatty acids affect key processes (e.g., inflammation, gap junctions protein expression, and enteroendocrine regulation) through G-protein coupled receptors, such as the GPR41 and GPR43 receptors, and favor the proliferation of certain bacteria species by maintaining an acidic pH [76,77]. Acetate, butyrate, and propionate induce the enteroendocrine L cells to release the YY peptide (PYY) and glucagon-like neuropeptide-1 (GLP-1). These peptides regulate digestion and modulate lipid metabolism, which indirectly affects fatty acid storage in the liver. Butyrate stimulates the intestinal epithelium, promotes GLP-2 release, and increases mucus secretion, reducing the intestinal barrier’s permeability. It also has anti-inflammatory properties and protects against colitis and colon cancer. Obesity metagenomics studies showed that SCFA pathways are activated, and SCFA levels are increased in overweight or obese subjects and animal models. Propionate as a gluconeogenesis substrate affects the central nervous system and protects the host against glucose intolerance and diet-induced obesity. Increased propionate levels are observed in microbiota after gastric bypass, which protect against diet-induced obesity when transferred to sterile mice [4,73].

In addition, the influence of microbiota on choline metabolism is currently under study, as microbiota is thought to synthesize trimethylamine (TMA) from dietary choline. Trimethylamine can be oxidized in the host liver by hepatic flavin-monooxygenase 3 (FMO3), producing trimethylamine *N*-oxide (TMAO) [4]. Circulating TMAO levels are associated with an increased risk of death from cardiovascular and cerebrovascular disease and type 2 diabetes mellitus (T2DM) [78,79]. Regulation of the gut microbiota activity, or the TMA-producing species, could help develop new ways to prevent or treat atherosclerosis and choline deficiency diseases. By reducing TMAO levels, resveratrol down-regulates the enterohepatic farnesoid X receptor (FXR):fibroblast growth factor 15 (FGF15) axis. It indicates that the gut microbiota can be targeted by personalized therapies, which can prevent or reduce the risk of metabolic diseases [80,81]. Another TMAO precursor, betaine, present in microorganisms, plants (e.g., bran, wheat germ, spinach), and seafood, protects cells and proteins from environmental stressors and serves as a source of methyl groups for transmethylation processes. Decreased levels of dietary methyl groups cause hypomethylation in several metabolic pathways, like hepatic fats metabolism, which leads to steatosis and plasma dyslipidemia. Betaine protects internal organs and reduces risk factors for vascular disease [81]. Moreover, berberine, an isoquinoline alkaloid extracted from herbal plants, inhibits choline-to-TMA conversion and alters gut microbiota composition, microbiome functionality, and gene abundance, and might become a potential future therapy for atherosclerotic disorders [82].

Ileum and colon microbiota produce secondary bile acids from cholesterol by cleavage of glycine and taurine and the -OH group. Secondary bile acids serve as signaling molecules by binding to receptors such as the G protein-coupled bile acid receptor 1 (TGR5), the vitamin D receptor (VDR), and the farnesoid X receptor (FXR) [83,84,85]. The ability to metabolize the naturally occurring FXR antagonist, tauro-β-muricholic acid, is the basic process leading to obesity, steatosis, and impaired glucose, insulin, and leptin tolerance. Importantly, the microbiota regulates immune processes at the tissue level through tryptophan metabolism. In particular, commensal *Lactobacilli* use tryptophan as an energy source to generate aryl hydrocarbon receptor (AhR) ligands and a transcription factor playing role in the organogenesis of intestinal lymphatic vesicles (ILF). AhR influences the production of IL-22 and, thus, influences the secretion of antimicrobial peptides (Lipocalin-2, S100A8, and S100A9) [86].

Other microbial metabolites, with yet unconfirmed functions in the physiology and pathophysiology of the host, include: indole propionic acid, bound to the intestinal epithelial barrier; ethylphenyl sulfate, correlated with enhanced autistic behavior in a mouse model [87]; indoxyl sulfate and p-cresyl sulfate, both associated with endothelial dysfunction and media arterial calcification in uremic patients [88].

The gut–brain axis plays a crucial role in preserving homeostasis. The gut microbiota communicates to the central nervous system through neuronal, endocrine, and immune pathways. Signals to the gut are modulated and transmitted through the vagus nerve and neuroendocrine pathways [89]. The way of communication with the microbiota can be direct when neurotransmitters—5-hydroxytryptamine (5-HT), γ-aminobutyric acid (GABA), and catecholamines are detected by microorganisms or indirectly by influencing the intestinal niche. The gut microconditions can be regulated by the vagus nerve involved in gut physiology, immune response, gut motility, and gut barrier function, all of which affect the composition and function of the microbiome [90]. The gut microbiota metabolites can act as signaling molecules that regulate the secretion of the enteroendocrine cells hormones: peptide YY (PYY) and glucagon-like peptide-1 (GLP-1). Glucagon-like peptide-1 and PYY have receptors in brain regions responsible for regulating the host’s energy balance [91].

## 6. A High-Fat, High-Protein Diet Effects on Gut Microbiota

High-protein diets are believed to promote weight loss and maintain healthy body weight in humans [92,93,94], but a systematic review of the literature on the subject showed that the long-term effects of protein-rich diet are neither consistent nor conclusive [95]. Studies conducted on rodents confirmed that a high protein-to-carbohydrate ratio prevents obesity induced by a high-fat diet [96,97,98,99,100,101,102,103,104,105].

The idea that the obesity-inducing potential of high-fat diets in murine models can be lowered by increasing the protein-to-carbohydrate ratio is largely based on research using casein or whey as a protein source. However, little is known about how different protein sources can modulate the response to high protein intake. Feeding obese-prone C57BL/6J mice with a high-fat and high-protein diet using beef, chicken, pork, cod fillets casein, or soy as protein sources leads to statistically significant differences in the obesity development under thermoneutral conditions. Casein most efficiently prevented weight gain and fat mass accumulation. At the same time, mice fed a high-protein diet based on ‘meat’ (pork, chicken fillets) showed the highest feeding efficiency and a moderate gain in adipose tissue mass [106]. Epidemiological studies also show that the consumption of dairy and vegetarian sources of protein is associated with protection against obesity, while a high consumption of meat, especially red meat, determines a more significant weight gain [107,108,109]. 

Martinez-Lopez et al. [110], using a canine model, demonstrated that the applied nutritional model altered the overall taxonomic composition of the microbiome. A high-protein diet correlated with the increase in bacteria belonging to the *Fusobacteria* and *Bacteroidetes* phyla, whereas a high-fiber diet with an increase in *Firmicutes* and *Actinobacteria* phyla [110]. In the study conducted on athletes, a 10-week consumption of protein supplement (10 g whey isolate and 10 g beef hydrolysate) was associated with a decrease in *Lachnospiraceae*, *Roseburia*, *Blautia*, *Synergistales*, *Coprococcus*, *Lactobacillales*, *Bacilli*, and *Bifidobacterium longum*, a higher abundance of *Bacteroidetes*, and lower abundance of *Firmicutes*, with no differences in microbial metabolites when compared with a placebo [111]. McKenna et al. [112] showed that high beef protein intake, combined with resistance training, is associated with a reduced abundance of *Veillonellaceae*, *Akkermansia*, and uncultured *Eggerthellaceae* and *Ruminococcaceae* [113]. On the other hand, Cronin et al. [113] reported no significant shift in the intestinal microbiome of overweight and obese patients supplementing whey protein, which may result from a relatively short duration of the dietary intervention. However, they noted an increase in trimethylamine *N*-oxide (TMAO) concentration [113]. Interestingly, consumption of the Mediterranean diet, rich in vegetal proteins and polysaccharides, was correlated with a higher total concentration of short-chain fatty acids (SCFA) in fecal samples [114].

Nutritional patterns are related to the genetic diversity of bacteria in the human gut [115]. In addition, dietary characteristics, such as fat content, whole grain content, fruit and nuts consumption, or a fiber-rich diet, affect the gut microbiota [9,10,12,13,14,15,116]. Nevertheless, which food components are particularly conducive to the growth and function of beneficial bacteria in the intestine is still not established. Rats fed with proteins derived from red meat (beef and pork), chicken and fish (here referred to as white meat), and other sources (casein and soybeans) showed differences in the gut bacteria profiles [117,118]. Research on this subject in humans is insufficient, and thus, the influence of the protein source and quality on the energy balance regulation needs further investigation.

Diets high in saturated- and trans-fats, known as Western-style diets, are believed to increase the risk of cardiovascular disease and atherosclerosis by increasing the total and LDL cholesterol in the blood [119]. On the other hand, health-promoting fats, such as monounsaturated and polyunsaturated fats, are vital in reducing the risk of chronic disease. The typical Western diet is rich in trans- and saturated-fats and low in mono- and polyunsaturated fats, predisposing ordinary consumers to develop metabolic syndromes, cardiovascular diseases, and obesity [120]. Several studies on patients have suggested that a high-fat diet increases the total anaerobic microbiota and the number of *Bacteroides* [115,121,122,123]. 

The Western-style diet induces alternations in gut microflora by promoting the abundance of *Proteobacteria*, *Mollicutes*, and *Bilophila wadsworthia,* increasing the *Firmicutes*-to-*Bacteroidetes* ratio, and reducing the abundance of bacteria considered as beneficial—i.e., *Akkermansia muciniphila*, *Bifidobacterium* spp., and butyrate-producing taxa [9,124,125]. In addition, the carbohydrates intake restriction is not without significance in high-fat diets, as the ketogenic diet contributes to the decreased gut colonization by *Bifidobacterium* and *Lactobacilli* and increased by *Fusobacteria* and *Escherichia* profusion [126].

Fava et al. [123] noted that a low-fat diet increased the fecal abundance of *Bifidobacterium* and reduced fasting glucose and total cholesterol. On the other hand, a diet high in saturated fat increased the relative proportion of *Faecalibacterium prausnitzii*. Finally, a high monounsaturated fat intake did not change the relative abundance of any bacteria, but decreased the overall bacterial load and plasma total cholesterol and LDL levels [123]. Similarly, the consumption of salmon—rich in mono- and polyunsaturated fats—also did not change the fecal microbiota composition in 123 people [127]. Studies in rats showed that a high-fat diet consumption reduced the amount of *Lactobacillus intestinalis* and the excessive growth of propionate- and acetate-producing species, including *Clostridiales*, *Bacteroides*, and *Enterobacteriales*. In addition, the number of *Lactobacillus intestinalis* negatively correlated with the rats’ body and adipose tissue weight [128]. Changes in the gut microbiota also influenced the development of inflammation induced by metabolic endotoxemia in mice consuming a high-fat diet [129]. The studies in mice comparing the effects of lipids derived from lard and fish oil on the gut microbiota showed that mice fed with lard presented with an increase in *Bacteroides* and *Bilophila*, while *Actinobacteria* (*Bifidobacterium* and *Adlercreutzia*), lactic acid bacteria (*Lactobacillus* and *Streptococcus*) and *Verrucomicrobia* (*Akkermansia muciniphila)* were more abundant in mice fed with fish. In addition, the lard-fed mice had an increased systemic Toll-like receptor (TLR) activation, white adipose tissue generalized inflammation, and impaired insulin sensitivity compared to fish oil-fed mice. The authors suggested that these findings result partly from differences in intestinal microbiota between the two groups; the transplantation of microbiota from one group to another after an antibiotic administration not only enriched the gut of the transplant recipient with the dominant types of the donor species, but also restored the inflammatory and metabolic phenotypes of the donor. These results suggest that the gut microbiota may promote metabolic inflammation through TLR signaling when challenged with a diet rich in saturated fats [130].

## 7. The Interaction of Metabolism and Diet in Relation to Gut Microbiota

The gut microbiota of traditional rural populations from different parts of the world showed greater bacterial diversity and the otherwise lacking microbial taxa compared to Western populations. Modern lifestyles, medical practices, and processed foods contribute to an overall decline in biodiversity and loss of specific phylogenetic groups from the gut microbiome of the industrialized populations. The gut microbiota modifies the bioavailability, transformation, absorption, or excretion of chemical elements (i.e., selenium, zinc, cobalt, and iodine) acting as cofactors for different enzymes involved in epigenetic modifications [131]. Yang et al. [132], studying the Chinese population, also proved the importance of environmental factors for gut microbiota diversity. They found that children from rural areas had an increased *Prevotella*-to-*Bacteroides* ratio, but a decreased abundance of microbiota when compared to children living in an urban environment [132]. Studies showed that host genetics and diet are related and can regulate the microbiome’s composition. Various genetic variants, especially those related to T2DM, obesity, dietary preferences, and metabolism, can significantly affect changes in obesity and the metabolic response of low-calorie weight loss diets [78]. A significant variant of the lactase gene that modulates *Bifidobacterium* sp. appears throughout the genome in the LCT region and is widespread among the intestinal microbiome of the dairy-consuming individuals. A study in the older Mediterranean population showed the LCT variant is associated with obesity and regulated by lactose and milk consumption [133]. Therefore, the diet can alter the microbial composition or abundance and the microbial metabolome [134,135]. Studies in Japanese populations revealed a transfer of the genes encoding the enzymes involved in the red sea algae metabolism from marine bacteria present in the dietary seaweed to the consumer’s gut microbiome specific bacteria [75]. The microbiome plays a key role in forming the hologenome, defined as the sum of the microbiota and host’s genetic information, resulting from their long-term co-evolution, ultimately defining the host’s metabolic capacity [136,137]. Many bacterial enzymes, like beta-glucuronidase, beta-glucosidase, azoreductase, nitroreductase, 7-alpha-dehydroxylase, and cholesterol dehydrogenase, are inducible diet-dependent enzymes. Many studies report on gene–diet interactions regulating *Bifidobacterium* sp. and other species’ abundance, indicating the importance of the host–microbiome interaction [70,134]. Microbiota can promote epigenetic modifications: the host responds to environmental factors by altering DNA methylation and modifying histones. DNA methylation influences gene expression by regulating the availability of transcriptional mechanisms, transcription factors, histone modifiers, and chromatin. DNA methyltransferases (DNMT) can add a methyl group from the S-adenosylmethionine donor (SAM) to the cytosine carbon-5 position (5 mC). At the same time, the dioxygenase family, ten-eleven translocation (TET) enzymes, oxidizes 5 mC to hydroxymethylcytosine (5 hmC). *Bifidobacterium* and *Lactobacillus* produce folic acid, which supports the production of S-adenosylmethionine (SAM) [138]. Dietary methionine modulates the composition of the host’s microbiota and the metabolism of the bacteria, releasing substrates for the synthesis of S-adenosylmethionine (SAM). The mechanisms of microbiota-dependent modification of histones are not well understood yet. Histone acetylation uncovers nucleosomal DNA targets for transcription factors, while histone deacetylation (HDAC) triggered deacetylation removes acetyl groups from histone tails, thus reducing transcriptional availability. Butyrate, produced by numerous commensal bacteria (i.e., *Faecalibacterium*, *Coprococcus*, *Roseburia*, *Eubacterium*) from dietary fiber, is an anti-inflammatory HDAC inhibitor by inhibiting the STAT1 and NF-kB activation [139].

Several infectious agents (Human papillomavirus, Hepatitis B and C viruses, Epstein–Barr virus, Polyomaviruses, *Chlamydia pneumoniae*, *Campylobacter rectus*, *Streptococcus bovis*, and *Helicobacter pylori*) and members of the intestinal microbiota are epigenetic factors involved in the pathogenesis of the metabolic syndrome. An example of the indirect action of small-molecule microorganisms (LMW) on chromatin remodeling is the deficiency of some substrates (betaine, methionine, choline) or cofactors (vitamins B12, B2, B6, and folic acid) produced by the microbiota. Gut indigenous bacteria can change the bioavailability of dietary methyl groups, causing hypomethylation of several epigenome-associated pathways. This change may hinder DNA methylation, leading to decreased SAM levels, increased plasma homocysteine levels, and an increased risk of various liver and vascular diseases and malignancies. LMW molecules, including SCFA, sulforaphane cysteine/sulforaphane *N*-acetylcysteine, and allyl mercaptan/diallyl disulfide produced during the metabolism of cruciferous vegetable or garlic microbes, may interfere with the activity of other enzymes responsible for the epigenetic modification, such as deacetylase, protein–threonferinase kinases, and syphosphyltransferinases. Additionally, the intestinal microbiota is the main donor of acetyl groups for the formation of acetyl-CoA, which is involved in epigenomic acetylation reactions. Bacteria and eukaryotes biosynthesize coenzyme A (CoA) from pantothenate, cysteine, and β-alanine, all of which are found in most foods in small amounts and are also produced by the gut microbiota. Deficiencies of these nutrients disrupt the synthesis of NADH, acetyl-CoA, and NAD, leading to disorders of the epigenomic mechanisms of acetylation involved in the change of chromatin structure and post-translational modifications of proteins [131].

## 8. Examples of Research Results

Endocannabinoids are endogenous signaling particles implicated in many physiological processes, including energy balance regulation, fat accumulation, and body homeostasis. They modulate the expression of insulin and adipokines through cannabinoid receptors, and their levels are known to be elevated in states of hyperglycemia and obesity [140]. Nevertheless, little is still known about the genetic and dietary factors affecting endocannabinoid system modulation. Ijaz et al. [141] investigated the impact of dietary proteins derived from casein, chicken, beef, and pork contained in a high-fat diet on endocannabinoids, adipogenesis, and biomarkers associated with dyslipidemia. A high-fat diet of beef or poultry increased the activity of the cannabinoid receptor 1, *N*-acylphosphatidylethanolamine-selective phospholipase-D, and diacylglycerol-α lipase in adipose tissue. The diets also decreased the immunoreactivity of the mitochondrial uncoupling protein 1 in brown adipose tissue. In addition, high-fat diets with beef and poultry protein had a significant effect on adipocyte differentiation and mitochondrial biogenesis in obese mice [141]. Sequencing of the 16S rRNA gene showed that high-fat diets significantly improved the ratio of *Firmicutes* to *Bacteroidetes* in the colon, regardless of protein source. Meat proteins in a high-fat diet significantly reduced the relative abundance of *Akkermansia* and *Bifidobacteria*, but increased the serum lipopolysaccharide levels, which promoted adipogenesis, causing endocannabinoid receptor dysregulation [141]. In animals with obesity, visceral obesity, and dyslipidemia, meat protein consumption reduced thermogenesis and affected mitochondrial activity compared to casein protein consumption. High-fat diets resulted in a significant increase in the number of *Firmicutes*, which was accompanied by a significant reduction in the number of *Deferribacteres* compared to animals fed with low-fat diets [141]. In animals fed with high-fat diets with chicken protein, *Verrucomicrobia* was the most common compared to others fed with high-fat diets, while in animals fed with high-fat diets with beef protein, *Verrucomicrobia* was the lowest [141]. In addition, there were more *Proteobacteria* in animals fed with a high-fat diet with beef protein than in the casein- and pork-protein-fed animals. The lean mice fed with casein had the greatest abundance of *Actinobacteria*, but its relative abundance remained somewhat similar to that of the obese mice. *Firmicutes*, *Bacteroidetes*, and *Verrucomicrobia* were the most abundant species in animals fed with low-fat diet groups. At the same time, a high-fat consumption increased the abundance of *Firmicutes*, *Proteobacteria*, and *Deferribacteres,* and decreased the abundance of *Verrucomicrobia*.

The essential role of microbiota on the intestinal endocannabinoid system was demonstrated in numerous studies. Oral administration of *Lactobacillus acidophilus* in mice and rats led to an increased expression of intestinal epithelial cannabinoid receptor 2 (CB2R) [142]. In addition, an increased consumption of oleic acid and omega-3 fatty acids, such as in the Mediterranean diet, resulted in significant increases in both the *N*-acyl-ethanolamines (NAEs) and 2-monoacyl-glycerols (2-MAGs), which are endocannabinoid congeners [143].

A high-fat diet increases the biodiversity of gut microbiota and is characterized by a high abundance of *Muribaculaceae*, *Rikenellaceae* RC9 gut group, *Odoribacter*, *Mucispirillum*, *Alistipes*, uncultured *Muribaculaceae* bacteria (two OTUs), and an uncultured *Bacteroidales* bacterium in comparison to a low-fat diet. In contrast, limited lipid consumption reduces gut microbial biodiversity in the colon but supports the retention of beneficial microorganisms.

The gut microbiome of animals fed with a low-fat diet consisted mainly of *Lactobacillus*, *Faecalibaculum*, *Lachnoclostridium*, *Bacteroides*, *Desulfovibrio*, *Eubacterium fissicatena* group, *Akkermansia*, and *Bifidobacterium* [144].

Compared to the casein diet group, the chicken, beef, and pork diet groups had a higher *Akkermanis* abundance, but a smaller *Faecalibaculum*, non-cultured *Lachnospiraceae*, *Blautia*, and *Lachnospiraceae* NK4A136 groups. High-fat diets significantly changed the composition of the gut microbiota, their biochemical environment and hence the microbial activity, in mice [145].

Ijaz et al. [146] studied the effects of different protein sources on microbial colon diversity in combination with a high- or low-fat diet. The gut of rodents fed with a low-fat diet were predominantly colonized by *Bacteroidales* S24-7, *Akkermansia*, *Rikenellaceae* RC9 gut group, *Desulfovibrio*, *Faecalibaculum*, *Alistipes*, and *Ruminiclostridium* 9. Consumption of a high-fat diet significantly reduced the abundance of the *Bacteroidales* S24-7 group compared to consumption of a low-fat diet. In addition, soy protein in a high-fat diet was associated with a relative abundance of *Proteobacteria* compared to beef protein or casein diets. Moreover, beef as a protein source crucially depleted the *Verrucomicrobia* abundance, regardless of the fat content in the diet. Relatively higher numbers of the genera linked with the development of obesity and the related metabolic syndrome, such as *Mollicutes*, *Oscillibacter*, *Escherichia*, *Shigella*, and *Mucispirillum*, were observed in animals fed with high-fat with beef proteins. Beef as a protein source in a high-fat diet excessively lowered the prevalence of *Akkermansia* by up to 23%. On the other hand, it affected the amount of *Blautia*, *Anaerotruncus*, and *Bacteroides*, which are inversely correlated with obesity and visceral obesity [146,147].

Zhu et al. [148] assessed the gut microbiota and metabolite composition in the distal colonic contents of rats fed with diets containing different protein sources: casein, beef, chicken, or soy for 90 days. The rats fed with chicken protein showed the highest relative abundance of *Lactobacillus* and the highest concentration of organic acids, including lactate, which may favor *Lactobacillus* growth, compared to rats fed with casein (control group). The rats fed with soy protein had the highest relative abundance of *Ruminococcus,* but the lowest relative abundance of *Lactobacillus* [148]. The long-term consumption of soy protein led to the upregulation of the transcription factor CD14 receptor and liver lipopolysaccharide-binding protein (LBP), which are involved in inflammatory processes and modulate macrophages activation [149]. These observations indicate that meat protein consumption could support a more balanced composition of gut microbiota, reduce the antigen load, and, consequently, lessen the inflammatory response of gut bacteria to the hosts. Contrary to meat proteins, a soy-based diet may enhance the degradation of dietary fiber and glycans, resulting in a higher SCFA production. Although higher concentrations of SCFAs were noted, the decreased relative abundance of beneficial bacteria was observed in rats fed with vegetable protein compared to rats fed with meat protein groups. Additionally, the rats fed with vegetable protein showed elevated levels of proteins associated with antioxidative stress response in the liver, which may suggest a shift of redox status toward pro-inflammatory processes [148]. In addition, the elevated glutathione S-transferase activity in the liver of these rats implied intensified detoxification and, thus, maintaining oxidative/antioxidative balance. 

The excessive consumption of red meat is associated with an increased risk of metabolic and cardiovascular diseases [150]. As numerous studies proved, increased red meat intake alters the composition and diversity of gut bacteria, favoring a greater abundance of *Fusobacterium* and *Bacteroides* and a lower abundance of *Lactobacillus* and *Roseburia* in the intestine. It was found that these changes were also accompanied by a significant reduction in the number of *Firmicutes* and *Bacteroidetes* [151]. However, Zhu et al. [117] noted a higher *Lactobacillus* but lower *Bacteroides* content in the meat protein-fed groups, while OTU227 (genus *Roseburia*) was found in the non-meat protein-fed than in the meat protein-fed group, but the relative abundance of *Roseburia* did not differ significantly between these two groups [117]. Meat protein-fed groups presented with a higher *Firmicutes* abundance and lower *Bacteroidetes* abundance than non-meat protein-fed groups [117].

Zhu et al. [117] also assessed the effects of different source proteins on the composition of cecal bacterial communities. They investigated the microbiota of rodents fed with red meat (beef and pork), white meat (chicken and fish), dairy (casein), and vegetable (soybeans) proteins at recommended levels of consumption. By sequencing the V4–V5 region of the 16S ribosomal RNA gene, they found that animals fed with meat proteins had a similar overall gut microbiota structure in the cecum to animals fed with non-meat proteins. The rodents fed with white meat protein showed a higher content of the *Lactobacillus* genus when compared to rodents fed with red meat or non-meat protein. Moreover, in contrast to rodents fed with vegetable protein, rodents fed with meat and dairy protein had significantly lower levels of lipopolysaccharide-binding proteins (LBP), suggesting that the consumption of meat proteins may maintain a more balanced composition of the gut bacteria, thus reducing the inflammatory response in the host [117]. 

However, it must be considered that the discussed studies have limitations. Consumption, preparation methods, and other ingredients of red meat, such as heme iron, can also affect the presented results and should be considered when assessing the relationship between meat or protein consumption and health problems. Overall, dietary proteins significantly influence the composition of the gut bacteria in the cecum. Specific phylotypes that respond to dietary proteins may play a key role in maintaining the host’s health. The consumption of dairy products and meat proteins at the recommended levels may support a balanced gut bacteria composition than soy protein [117]. These findings provide new insight into the relationship between meat consumption and gut bacteria health and indicate that low red- or white-meat protein consumption may be more beneficial to health than non-meat proteins consumption [117].

Table 1 presents the summary of research on the effects of dietary fats and proteins on the gut microbiome.

## 9. Conclusions

While the effect of diet on the gut microbiota is well understood, further information on the effects and duration of action of specific dietary components remains unexplored. The analysis of the nutritional models’ impact on the intestinal microbiota condition may allow for developing the optimal dietary programs based on the consistently and selectively supplied nutritional substrates to the environment of intestinal microorganisms and the implantation and proliferation of bacterial cultures. The use of acute dietary interventions in humans leads to transient changes in the intestinal microbiota from over days to several weeks. Knowledge of how eating habits and diet—including animal products—affect the gut microbiota in the long term is limited by the lack of long-term nutritional studies or interventions repeating over multiple time points. In addition, the variety of available research results makes it difficult to view and analyze the factors influencing the diet–microbiota relationship, possibly due to the personalized responses of the host’s microbiota.

Further long-term dietary interventions and observations, including nutrient analysis, are needed to investigate the potential of the diet-induced gut microbiome. For this, a wide range of individual microbial profiles and personalized therapeutic strategies must be considered.

Personally-tailored therapies based on the individual composition of the intestinal microbiota and the possibility of its disruption may become the basis for treating metabolic disorders in the future.

## Figures and Tables

**Table 1:** The summary of research on the effects of dietary fats and proteins on the gut microbiome in human, rodent, murine, and canine models.

Research Model	Timeframe	Protein or Fat Source	Microbiota Changes	Comment	Ref.
Human N = 27		Mediterranean diet	greater *Bacteroidetes* presence associated with lower animal protein intake		[114]
			higher *Bifidobacterium* spp. levels associated with more vegetal proteins intake; lower *Bacteroidetes* presence and higher *F/B* ratio associated with higher animal protein intake		
			lower relative abundance of *Parabacteroides* and *Butyricimonas* related to higher animal proteins and saturated fats intake; lower relative abundance of *Oscillospira* related to high protein intake; *Roseburia* associated with vegetal proteins intake		
Human, male N = 24	10 weeks	whey isolate (10 g) and beef hydrolysate (10 g) vs. maltodextrin; once a day	protein group presenting higher abundance of *Bacteroidetes* and lower abundance of *Firmicutes*		[111]
			protein group presented a higher percentage of *Bacteroides* genus and a lower presence of *Citrobacter* and *Klebsiella* genera		
Human, overweight N = 50	10 weeks	isocaloric meal of minimally processed beef (97.4% lean): moderate protein consumption (16 g protein, MOD) vs. high protein consumption (32 g protein, HIGH) of the minced beef steak	1st week of dietary habituation: decreased abundance of *Veillonellaceae*, *Akkermansia*, *Eggerthellaceae*, and *Ruminococcaceae* in the HIGH group; Final result: *Erysipelotrichaceae* decreased in MOD and HIGH groups, increased abundance of *Eggerthellaceae*, *Veillonellaceae*, and *Akkermansia* in HIGH group, increased abundance of *Veillonellaceae* in MOD group	whole body resistance training (3 days/week) during the diet intervention	[112]
Human, predominantly overweight or obese, N = 90	8 weeks	daily 30-g protein supplement containing 24 g of whey protein (P group)	increased alpha diversity of *Archaea* sp. in the P group; moderately enhanced archaeal diversity in the P group compared to the EP group; greater bacterial diversity in the EP group than in the P group	P group vs. EP group (exercise + diet of P group) vs. E group (exercise only)	[113]
			E and EP groups with separate *Prevotella copri*-related clusters; P group composed of *Bacteroides vulgatus* pathways; no significant separations for alterations in diversity resulting from the intervention (P, EP, E)		
Human, pregnant women N = 123	ca. 18 weeks	two 150 g portions of farmed salmon/week	no effects of increased oily fish consumption on any of the bacteria enumerated in maternal fecal samples	sampled at 38 weeks gestation	[127]
Rat, Sprague-Dawley, male N = 66	90 days	proteins from pork, beef, chicken, fish, soy, or casein	chicken and fish proteins-fed groups presented higher *Firmicutes* but lower *Bacteroidetes* abundance than groups fed with other proteins; soy protein-fed group presented higher *Bacteroidetes* abundance; chicken protein-fed group presented greater *Actinobacteria* abundance; beef protein-fed group presented greater *Proteobacteria* abundance		[117]
			*Lachnospiraceae* characteristic of soy protein and casein-fed groups (average: 17% and 18%, respectively); *Ruminococcaceae* (average: 18% and 27%, respectively) and *Lactobacillaceae* (average: 20% and 19%, respectively) characteristic of beef and pork proteins-fed groups; *Lactobacillaceae* (average: 46% and 36%, respectively) characteristic of chicken and fish proteins-fed groups		
			36 OTUs difference between non-meat and red meat protein groups: 22 OTUs higher in non-meat protein groups and 14 OTUs higher in red meat protein groups; relative abundance of *Alloprevotella* higher in non-meat protein groups, *Roseburia* one of the most predominant in non-meat protein groups, *Prevotellaceae* uncultured detected in non-meat protein groups but not in red meat protein groups		
			56 OTUs difference between non-meat and white meat protein groups: 33 OTUs higher in non-meat protein groups, 23 OTUs higher in white meat protein groups; *Roseburia* and *Prevotellaceae* uncultured typical of non-meat protein groups; *Bacteroides* characteristic of non-meat protein groups; 5 OTUs representing genus *Lactobacillus* more abundant in white meat protein groups		
			105 OTUs difference between red and white meat protein groups: 83 OTUs higher in red meat protein groups; 22 OTUs higher in white meat protein groups but only 16 OTUs significantly diverse; relative abundance of *Lactobacillus* genus higher in white meat protein groups; relative abundance of *Oscillibacter* was higher in red meat protein groups; *Bacteroides* differed between the red and white meat protein groups; chicken protein-fed group with the highest, and casein-fed group with the lowest *Lactobacillus* abundance (multiple comparison); soy protein-fed group with lower *Lactobacillus* abundance compared to meat proteins-fed groups		
Rat, Sprague Dawley, 6-week old, male N = 20	16 weeks	control chow (11 kJ/g, 12% fat, 21% protein, 65% carbohydrate), free choice HFD in 3 variants: (1) control chow (2) commercial HF pelleted diet SF03-020 (20 kJ/g, 43% fat, 17% protein, 40% carbohydrate) (3) modified chow (powdered chow with sweetened condensed milk and saturated animal fat (lard); 15.4 kJ/g; 51% fat, 10% protein, 38% carbohydrate)	HFD decreased abundance of *Lactobacillales* and lowered abundance of *Clostridiales*, *Bacteroidales*, *Enterobacteriales*, *Erysipelotrichales* and *Desulfovibrionales*	fecal sample/animal; harvested from the terminal part of the cecum	[118]
			HFD lowered abundance of *Lactobacillaceae* and increased abundance of other important groups including *Bacteroidaceae*, *Lachnospiraceae*, *Enterobacteriaceae*		
			*Ruminococcaceae*, *Veillonellaceae*, *Porphyromonadaceae,* and *Erysipelotrichaceae*		
			*Lactobacillus*, specifically *Lactobacillus intestinalis*, dominated in chow-fed groups; HFD decreased *Lactobacillus intestinalis* but increased *Blautia*, *Morganella*, *Bacteroides*, *Phascolarctobacterium,* and *Parabacteroides*		
Rat, Sprague-Dawley, male N = 32; n = 8 per group	90 days	casein, beef proteins, chicken proteins, soy proteins	no differences in ACE, Chao, Shannon, Simpson, and Good’s coverage indices; different response to chicken protein than to casein, beef protein, and soy protein; soy and casein-fed groups showed similarity in microbiota; *Firmicutes* and *Bacteroidetes* the most dominant phyla; *Bacteroidetes* the most and *Firmicutes* the least abundant in chicken protein-fed group; *F/B* ratio was lower in casein, beef, and soy protein-fed groups		[148]
			*Fusobacterium* higher in casein and beef protein-fed groups; chicken protein-fed group with the highest relative abundance of *Lactobacillus* sp. OTUs and higher level of beneficial *Lactobacillus* sp.; soy protein-fed group with the highest relative abundance of *Ruminococcaceae* OTUs and the lowest level of beneficial *Lactobacillus* sp.		
Mouse, 12-week-old, male	11 weeks	lard vs. fish oil	*Bacteroides*, *Turicibacter*, and *Bilophila* higher in lard-fed group; *Actinobacteria* (*Bifidobacterium* and *Adlercreutzia*), lactic acid bacteria (*Lactobacillus* and *Streptococcus*), *Verrucomicrobia* (*Akkermansia muciniphila*), *Alphaproteobacteria*, and *Deltaproteobacteria* higher in fish-oil-fed group	gut microbiota transplantation with cecal content followed by 200 μL antibiotic cocktail treatment (ampicillin + metronidazole + vancomycin + neomycin) administrated by oral gavage once a day for 3 days	[130]
			*Akkermansia* and *Lactobacillus* presence increased in the cecal contents of fish oil-fed group compared to lard-fed group; *Lactobacillus*, but not *Akkermansia*, presence increased in fish-oil-fed group after 3 weeks; *Akkermansia* taxa increased in the cecum of mice that received fish-oil microbiota; *Lactobacillus* increased in mice that received a lard microbiota after gut microbiota transplant		
Mouse C57BL/6J, 7-week old, male N = 80	14 weeks	high-fat diet (HFD, 60% kcal from lard) vs. low fat diet (LFD, 12% kcal from lard) containing casein, or meat proteins from chicken, beef, or pork	Dietary proteins had no effect on microbiota richness (Chao and Good coverage) or the diversity (Shannon and Simpson indices) in LFD groups; beef and pork protein diet groups with lower Chao and Good coverage values than casein diet group among HFD groups		[141]
			HFD increased *Firmicutes* and reduced *Deferribacteres* abundance compared with LDF groups; HF chicken protein diet group with the highest, and HF beef protein diet group with the lowest, *Verrucomicrobia* abundance compared with other HFD groups		
			HF beef protein diet group with higher *Proteobacteria* abundance than casein and pork protein diet groups; LF casein diet group with the highest *Actinobacteria* abundance, but not the relative *Actinobacteria* abundance, remained similar to HF group; *Firmicutes*, *Bacteroidetes*, and *Verrucomicrobia* the most abundant in LF diet groups; HFD increased the abundance of *Firmicutes*, *Proteobacteria*, and *Deferribacteres* but reduced the abundance of *Verrucomicrobia*; *Bacteroidales* S24-7, *Akkermansia*, *Desulfovibrio*, *Rikenellaceae* RC9 gut group, *Faecalibaculum*, *Alistipes*, and *Ruminiclostridium* 9 the most abundant in LFD groups		
			chicken, beef, and pork protein diet groups with higher abundance of *Akkermanisa* but lower abundances of *Faecalibaculum*, *Lachnospiraceae* uncultured, *Blautia*, and *Lachnospiraceae* NK4A136 group than the casein diet group; HFD increased the relative abundances of *Desulfovibrio*, *Lachnospiraceae* uncultured, *Ruminiclostridium* 9, and *Lactobacillus*, but decreased the relative abundance of *Akkermansia*; HF beef protein group with relatively higher abundances of genera *Mollicutes*, *Oscillibacter*, *Escherichia*, *Shigella*, and *Mucispirillum* and decreased relative abundances of *Blautia*, *Anaerotruncus*, and *Bacteroides*		
			*Corynebacteriaceae*, *Micrococcaceae*, *Actinobacteria*, *Staphylococcaceae*, and *Lactobacillales* the most abundant taxa in LF casein diet group; *Peptococcaceae*, *Sphingomonadaceae*, *Burkholderiaceae*, *Pseudomonadaceae*, and *Anaeroplasmataceae* dominant in the chicken protein diet group; *Defluviitaleaceae* and *Verrucomicrobiaceae* more abundant in LF beef protein diet group; *Deferribacteraceae* and *Lactobacillales* rich in LF pork protein diet group; *Peptococcaceae*, *Ruminococcaceae*, *Clostridia*, *Alcaligenaceae*, and *Halomonadaceae* the most abundant in HF casein diet group; *Lactobacillales*, *Bacilli*, *Christensenellaceae*, and *Clostridialesvadin* bb60 group more specific for HF chicken protein diet group; *Porphyromonadaceae*, *Peptostreptococcaceae*, and *Burkholderiaceae* specific for HF beef protein diet group; *Rhodospirillaceae* specific for HF pork protein diet group		
Mouse C57BL/6J, male	14 weeks	high-fat diet (HFD, lard) vs. low-fat, high-carbohydrate diet (corn starch) (LFD)	no difference in *Firmicutes* abundance or *F/B* ratio between HFD and LFD groups; relative abundances of *Lactobacillus*, *Faecalibaculum*, *Lachnoclostridium*, *Bacteroides*, *Desulfovibrio*, *Eubacterium fissicatena* group, and *Bifidobacterium* higher in LFD group; *Lachnospiraceae*, *Blautia*, *Rikenellaceae* RC9 gut group, *Oscillibacter*, an uncultured *Bacteroidales bacterium*, *Lachnospiraceae* UCG-006 more abundant in HFD group; *Rikenellaceae* RC9 gut group, *Rikenellaceae*, *Clostridiales*, and *Peptococcaceae* higher in HFD group; *Lactobacillae* more abundant in LFD group	cecum	[144]
			alpha-diversity higher in the HFD group; relative abundances of *Bacteroidetes* and *Proteobacteria* higher in HFD group; relative abundances of *Firmicutes* and *Verrucomicrobia* lower in HFD group; *F/B* ratio significantly higher in LFD group	colon	
			HFD increased alpha diversity in cecum and colon compared to LFD; *F/B* ratio significantly decreased in HFD group HFD group: *Desulfovibrionaceae* bacterium the most abundant in cecum; uncultured *Muribaculaceae* bacterium was the most abundant in colon LFD-fed group: *Lactobacillus* was the most abundant in cecum and colon		
			*Muribaculaceae*, *Rikenellaceae* RC9 gut group, *Odoribacter*, *Mucispirillum, Alistipes*, uncultured *Muribaculaceae* bacteria (two OTUs), and an uncultured *Bacteroidales* bacterium more abundant in HFD group; *Faecalibaculum*, *Blautia*, *Bifidobacterium*, *Akkermansia*, and uncultured *Muribaculaceae* bacterium less abundant in HFD group; *Muribaculaceae* was more abundant in HFD group in the cecum and colon; HFD increased *Mucispirillum* in cecum and colon; *Lactobacillus* and *Bifidobacterium* abundance decreased in HFD group		
Mouse C57BL/6NCrl, male n = 6 per group	12 weeks	carbohydrate (corn starch) vs. high-fat (HF, beef tallow)	HF diet did not affect taxa richness; *Ruminococcaceae* (phylum *Firmicutes*) proportionally lower and *Rikenellaceae* (phylum *Bacteroidetes*) proportionally higher in HF-fed group; *Lactobacilli* in higher proportions in HF-fed group; HF-fed group with increased relative *Rikenellaceae* abundance; mean *Lactobacillus* relative abundances higher (but not statistically significant due to inter-individual variations) in HF-fed group		[145]
Mouse, C57BL/6J, male N = 60	12 weeks	low-fat diet (LFD: 12% kcal from lard) vs. high-fat diet (HFD: 60% kcal from lard); each diet with different protein source: casein (C), soy (S), beef (B)	no differences in HFD and LFD groups in alpha diversity; microbiota responded differently to beef protein in LFD and HFD groups HFD increased *F/B* ratio compared to LFD; *Firmicutes*, *Bacteroidetes*, and *Verrucomicrobia* the most abundant in LFD and HFD groups		[146]
			relative abundances of *Firmicutes* and *Bacteroidetes* unchanged in all HFD groups in soy, casein, and beef protein-fed subgroups; *Verrucomicrobia* abundance reduced in HFB group compared with LFB group and other HFD groups; relative abundance of *Proteobacteria* higher in HFS group than in HFB and HFC groups		
			*Bacteroidales* S24-7 the most predominant genus in LFD-fed groups; HFD reduced the abundance of *Bacteroidales* S24-7 group compared with LFD; HFD increased the abundance of *Mucispirillum*, *Escherichia*, *Shigella*, *Mollicutes*, and *Oscillibacter* and their relative abundances were highest in HFB group; HFB reduced the relative abundance of *Akkermansia* but induced an increase in relative abundance of *Anaerotruncus*, *Bacteroides*, and *Blautia*; LFS-fed group with higher relative abundance of *Rikenellaceae* than LFB-fed group; *Akkermansia* was most abundant in LFB-fed group and least abundant in LFC-fed group; LFB increased the relative abundances of *Mucispirillum*, *Deferribacteraceae*, *Desulfovibrionaceae*, and *Bacteroidaceae* LFC group showed the highest relative abundances of *Firmicutes*, *Actinobacteria*, *Bacilli*, and *Lactobacillus*, but lower relative abundances of *Akkermansia*, *Deferribacters*, and *Ruminiclostridium*		
			*Lachnospiraceae* NK4A136 group the most predominant in HFS-fed group and the least abundant in the HFB-fed group; HFB-fed group with the highest relative abundances of *Blautia*, *Romboutsia*, and *Odoribacter*; HFC-fed group with the highest relative abundances of *Ruminiclostridium* 9, *Lactobacillus*, *Anaerotruncus*, and *Actinobacteria*		
Dog N = 50	18 weeks	diet A = hydrolyzed diet (protein source: hydrolyzed chicken liver, carbohydrate source: corn starch and cellulose) diet B = high-insoluble fiber diet (protein source: soybean meal, carbohydrate source: soybean meal) diet C = high-protein diet (all meat/carcass, raw diet)	*Firmicutes* represented 44% (range: 18–91%) in diet C, 62% (range: 29–93%) in diet B, and 55% (range: 30–95%) in diet A; *Bacteroidetes* represented 14% (range: 0.22–50%) in diet C, 16% (range: 0.44–41%) in diet B, and 16% (range: 0.34–51%) in diet A; *Fusobacteria* represented 24% (range: 4–72%) in diet C, 8% (range: 1–45%) in diet B, and 17% (range: 2–34%) in diet A	Group 1 = dogs fed ACB diet sequence Group 2 = dogs fed BCA diet sequence each feeding period = 6 weeks; all dogs fed with diet C at baseline	[110]
			after 6 weeks on diet A, relative abundance of *Bacteroidetes* was 24% (range: 0.71–51) in group 1 and 7% (range: 0.34–29) in group 2; *Bacteroidetes* presented 3–50% (median: 23%) at baseline (diet C) in group 1 and 0.5–33% (median: 8%) at the end of the washout (diet C) period in group 2		
			diet C enriched with *Fusobacteria* in group 1; diets B and A enriched *Firmicutes* phylum; *Firmicutes* increased in the washout period but not during the baseline; *Bacteroidetes* increased at baseline but not during the washout period; *Actinobacteria* increased on diet B only in group 1		
			*Turicibacteraceae*, *Lactobacillaceae*, *Bifidobacteriaceae* and *Erysipelotrichaceae* higher on diet B only in group 1; *Peptostreptococcaceae* and *Clostridiaceae* higher on diet C only during the washout period; *Bacteroidaceae* higher only at baseline; *Fusobacteriaceae* more abundant at baseline and during the washout period in both groups; *Veillonellaceae* was more abundant on diet A only in group 1 and on diet B; *Prevotella* to *Bacteroides* ratio higher on diet A and B compared to diet C		

Abbreviations: *F/B*—*Firmicutes*-to-*Bacteroidetes*, OUT—operational taxonomic unit.

## Data Availability

There is no data created for this paper.
